# A Rare Case of Primary Pulmonary Meningioma

**DOI:** 10.3390/jcm14082688

**Published:** 2025-04-15

**Authors:** Calista Sha, Leo Li, Fernanda Mitchell, Frank Breuer, Riona Park, Paul C. Lee

**Affiliations:** 1 Department of Thoracic Surgery, Northwell Health Long Island Jewish Medical Center, New Hyde Park, NY 11040, USA; csha@northwell.edu (C.S.); lli7@northwell.edu (L.L.); rionahpark@gmail.com (R.P.); 2 Department of Pathology, Northwell Health North Shore University Hospital, Manhasset, NY 11030, USA; fmitchell@northwell.edu (F.M.); fbreuer@northwell.edu (F.B.)

**Keywords:** primary pulmonary meningioma, wedge resection, lung cancer, lung nodules

## Abstract

**Background**: Primary pulmonary meningioma (PPM) is an uncommon tumor originating in the lung. Although predominantly benign, there are instances of aggressive tumors exhibiting malignant features. Due to their rarity, our understanding of PPMs is primarily gleaned from case reports or small case series. **Methods**: This report details the case of an 84-year-old female presenting with an incidental, well-circumscribed, enlarging nodule (1.4 × 1.3 × 0.9 cm) in the left upper lobe (LUL). Initial imaging found it to be highly suspicious of lung cancer. **Results**: Upon surgical resection, pathological analysis confirmed the tumor’s characteristics to be consistent with a benign PPM. Postoperative recovery was uneventful and there is no evidence of recurrence. **Conclusions**: Our report aims to contribute to the expanding body of knowledge concerning incidental PPMs by documenting our clinical encounter with this patient.

## 1. Introduction

A meningioma is a common type of intracranial tumor, constituting approximately 30% of all brain tumors. They originate from meningothelial or arachnoid cells from the meninges, the membrane that surrounds the brain and spinal cord [1]. Meningiomas are typically benign, and they rarely metastasize extracranially [2]. Therefore, a primary case of meningioma located in the lung is extremely uncommon. There are only about 70 reports of primary pulmonary meningiomas (PPMs) in the English literature between the years of 1982 and 2021. Cases mainly occur in patients between the ages of 40 and 60, and its prevalence is slightly higher in women than men [3]. This report details the case of an 84-year-old asymptomatic female in whom a PPM was incidentally discovered. The patient successfully underwent a thoracoscopic pulmonary wedge resection, and histopathological examination confirmed the diagnosis of benign PPM. Given the limited knowledge we have on PPMs, our case contributes to a deeper understanding of PPMs and underscores the importance of considering PPM as a potential diagnosis when encountering incidental pulmonary nodules, thus aiding in better patient management and treatment strategies.

## 2. Case Report

**Figure 1:**
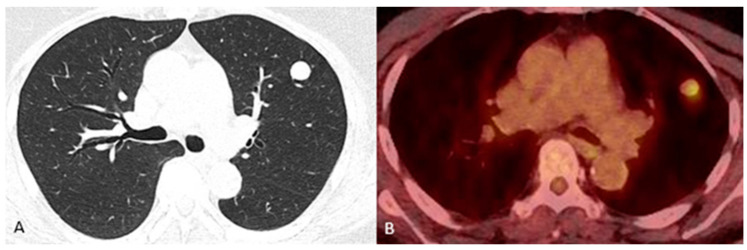
(**A**) Chest CT without contrast. (**B**) Fused PET and CT of an 84-year-old female diagnosed with a primary pulmonary meningioma. CT = computed tomography. PET = positron emission tomography.

An 84-year-old never-smoking woman with a history of type 2 diabetes mellitus, arthritis, and hypertension presented to our clinic with an incidental finding of an enlarging solitary lung nodule. She denied shortness of breath, cough, chest pain, hemoptysis, palpitation, fever, and recent illness. This nodule was being followed by the patient’s primary care provider, and it measured 0.9 × 0.8 cm (cm) 4 years prior. However, on her recent chest CT scan, this lesion was found to be 1.3 × 1.2 cm and well circumscribed. A positron emission tomography (PET/CT) scan revealed this nodule to be 1.4 cm with an avidity of 3.8 without any metastatic or nodal involvement (Figure 1). A head CT without contrast revealed mild white matter hypoattenuation, but no evidence of acute hemorrhage, mass effect, or extra-axial collection. The imaged portions of the skull, mastoid air cells, nasopharynx, and parotid gland are unremarkable. The initial impressions were that there was probable mild chronic small-vessel white matter ischemia, but evidence of any meningioma or suspicious nodule was not found in the brain.

The malignancy of our patient’s lung nodule was assessed with a whole-body PET/CT scan. There was no distant metastasis observed, and so addressing the isolated lung nodule was prioritized. Considering that the nodule was greater than 0.8cm in size, the cutoff size for a diagnostic biopsy, and the nodule’s increase in size over the preceding four years, the surgical team offered a diagnostic and therapeutic approach through surgery. A robotic approach was chosen due to the surgeon’s preference. Based on the preoperative assessment, differential diagnoses included carcinoid tumor, non-small-cell lung cancer, or a hamartoma. The patient then underwent a robotic-assisted left VATS wedge resection of the left upper lobe under general anesthesia to remove the nodule.

**Figure 2:**
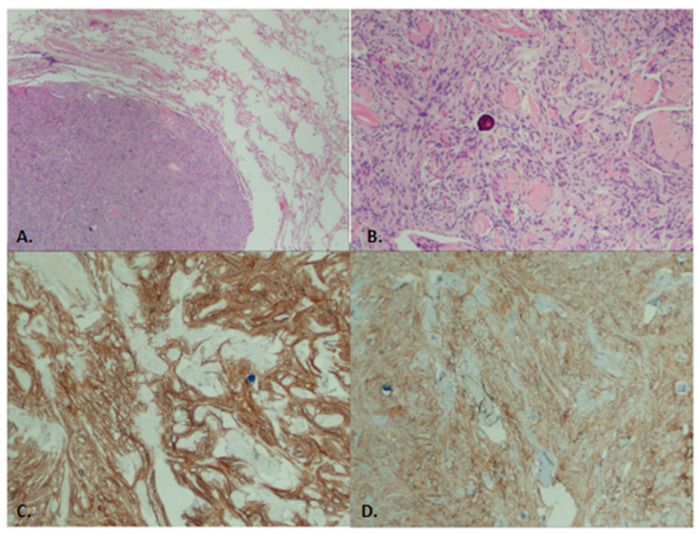
(**A**,**B**) Microscopic pathology of the surgical specimen (wedge) stained with Hematoxylin and eosin. (**A**) Meningioma mass adjacent to benign lung parenchyma—10× magnification. (**B**) Meningioma with whirling and psammoma bodies—10× magnification. (**C**,**D**) Immunohistochemistry evaluation. (**C**) Positive SSTR2—4× magnification. (**D**) Positive EMA—4× magnification.

Intraoperative pathological examination of our patient’s frozen sections revealed a well-circumscribed nodule with negative margins consistent with the diagnosis of intrapulmonary meningioma. Further pathological examination revealed a solitary, well-circumscribed nodule adjacent to unremarkable lung parenchyma (Figure 2A). The morphological features present were meningoepithelial cells organized in a syncytial pattern with invisible cell borders. The nuclei are small, oval, and irregular with fine powdery chromatin, inconspicuous nucleoli, and eosinophilic cytoplasm. Meningiomas often have whorls, which are spindle-shaped streams of nuclei, and psammoma bodies, both of which were present in our case (Figure 2B). The latter ones are calcifications with a laminated, concentric appearance.

Immunohistochemical staining and special stains confirmed the diagnosis of PPM. They showed positive staining for several markers, including Somatostatin Receptor 2A (SSTR2) and epithelial membrane antigen (EMA) (Figure 2B,C), all of which are commonly associated with meningiomas. In contrast, negative stains were observed for markers that are typically associated with other types of tumors, such as STAT6, Chromogranin, CAM5.2, p40, Estrogen Receptor (ER), Synaptophysin, Smooth Muscle Actin (SMA), Desmin, SOX-10, and CD34. These negative results helped rule out other potential diagnoses, reinforcing the diagnosis of PPM.

The patient’s recovery following surgery was uneventful, and she was discharged home on postoperative day one in stable condition. Six months after surgery, she reported experiencing left hip pain that radiated down her left leg, which is being investigated further. However, she denied any symptoms such as fever, chills, hemoptysis, lightheadedness, or dizziness, and her overall health has remained stable. Given her complaint of hip pain, she was referred to a pain management specialist for evaluation and appropriate management. Importantly, her follow-up imaging and clinical assessments continue to show no evidence of recurrence or metastasis 2 weeks, 1 month, and 6 months postoperatively. The patient will be closely monitored with regular follow-up visits, and further interventions will be considered if necessary.

## 3. Discussion

About 30% of all computed tomography (CT) scans contain an incidental pulmonary nodule, and of all lung nodules found on CT scans, 96% are benign [4,5]. Therefore, small incidental pulmonary nodules are usually insignificant and indolent. However, early-stage lung cancers are largely asymptomatic, so depending on the patient’s risk factors and the size and growth of the nodule, further clinical investigation may be worthwhile. The incidental lung nodule found on our patient’s chest CT scan was increasing in size; thus, it was suspicious of lung cancer. Upon surgical resection and intraoperative pathological and histological review, the lung nodule was confirmed to be a primary pulmonary meningioma.

Meningiomas can arise in any part of the lining over the brain and spinal cord and are most often found in the intracranial spaces [6]. While the majority of meningiomas are benign, there are rare cases where they metastasize extracranially [2,7]. Even rarer are incidences of primary extracranial central nervous system meningiomas [8,9].

Histologically, PPM is a benign tumor and appears as a solitary well-defined mass. Its basic cytologic features are spindled cells organized in bundles and whorls, with small, oval, and irregular nuclei with fine powdery chromatin, inconspicuous nucleoli, and eosinophilic cytoplasm. The presence of psammoma bodies always raises a red flag for a diagnosis of meningioma. In benign brain tissues, psammoma bodies are distributed within the connective tissue cores of the choroid plexus.

Despite first being reported in 1982 by Kemnitz and Heinrich, the exact origin of PPMs remains uncertain, with ongoing debate among researchers regarding the pathophysiological mechanisms behind their development [8]. Most cases of PPM, like other lung lesions, are asymptomatic. However, in cases where PPMs grow larger, patients may begin to experience symptoms of dyspnea and chest pain, due to the mass effect of the tumor on surrounding structures [9]. X-rays and CT scans of patients with PPM usually reveal round or oval solitary nodules, which can vary in size and appearance depending on the specific characteristics of the tumor and its location within the lung [10]. In most cases, PPMs are discovered incidentally during imaging studies performed for other clinical reasons. Often, the unknown lesion is treated as a primary lung cancer. Head CTs can be used to rule out brain metastasis, and a PET/CT can be used to rule out lymph node involvement and distal metastasis [11]. While head MRIs may be preferred over head CTs, our patient’s case had low suspicion for brain involvement and a head CT was deemed sufficient to rule out primary brain meningioma.

Given the well-defined boundaries of PPMs, surgical resection remains the preferred treatment strategy for managing these tumors [12]. In particular, Incarbone et al. have suggested that pulmonary wedge resection is often sufficient for benign PPMs, and that more extensive procedures such as lobar or sublobar resections are generally unnecessary in these cases [13]. This approach is further supported by a case study involving a patient with multiple benign PPMs who underwent successful surgical resections and remained disease-free for over a decade following the procedure [14]. However, there are also reports of malignant forms that demonstrate more aggressive clinical behavior. Pathological risk features can include a loss of the normal architectural pattern of the lung tissue, nuclear pleomorphism, an increase in mitotic activity, and the presence of prominent nucleoli [15]. One such case, described by Prayson et al., involved an aggressive PPM [16]. After the initial lobectomy of the right upper lobe, which removed a 6.5 cm nodule, the patient returned with complaints of shortness of breath. The patient underwent a complete pneumonectomy for a recurrent tumor in his right middle and lower lobes. Four months later, he returned with a 4.5 cm mass within the right lobe of the liver chest wall. While the recurrence of pulmonary meningioma after surgical resection is uncommon, extrapulmonary recurrence is even more so, highlighting the extreme malignancy of this case. Immunohistochemistry analysis revealed positive staining for vimentin, epithelial membrane antigen, and progesterone receptors. An MIB-1 labeling index of 9.2% was observed. Consistent with other intracranial lesions, cell proliferation markers, like MIB-1, are typically higher in recurrent and malignant tumors [17,18].

## 4. Conclusions

In conclusion, PPMs are rare, and their clinical trajectories are not well understood, posing challenges in diagnosis and management. While the majority of PPMS are benign, there have been documented cases of malignant PPMs in the literature, highlighting the importance of thorough evaluation when these nodules are detected. Due to the limited number of cases and the diverse nature of PPMs, determining their clinical course can be difficult, necessitating individualized approaches to treatment. As demonstrated in the case of our patient, when feasible, surgical resection, such as thoracoscopic pulmonary wedge resection, can serve both as a diagnostic tool and as a therapeutic intervention. This approach allows for definitive histopathological evaluation, confirming the nature of the nodule, and offers the potential for complete removal of the tumor, which can significantly improve patient prognosis.

## Data Availability

Data are unavailable due to privacy or ethical restrictions.
